# The effects of brines relevant to Mars and the ocean worlds on bacterial growth reflect salt-specific responses across water activity

**DOI:** 10.1007/s00203-025-04418-9

**Published:** 2025-09-09

**Authors:** Hassan Z. Zbeeb, MD Joad, Hadi H. Zayed, Ammar Mahdi, Fei Chen, Benton C. Clark, Thomas M. Luhring, Mark A. Schneegurt

**Affiliations:** 1 Department of Biological Sciences, Wichita State University, 26, 1845 Fairmount, Wichita, KS 67260 USA; 2 Jet Propulsion Laboratory, California Institute of Technology, Pasadena, CA 91109 USA; 3 Space Science Institute, Boulder, CO 80301 USA

**Keywords:** Astrobiology, Hypersaline, Extremophiles, Water activity

## Abstract

Freezing point depression due to high salt concentration is crucial for liquid water to exist on cold worlds, expanding special regions where habitats are plausible. Determination of the growth tolerances of terrestrial microbes in analog systems impacts planetary protection protocols aimed at preventing interference with life detection missions or potential native ecosystems on celestial bodies. We measured the salinity tolerances of 18 salinotolerant bacteria (*Bacillus*, *Halomonas*,* Marinococcus*,* Nesterenkonia*, *Planococcus*, *Salibacillus*, and *Terribacillus*). The salts tested to high concentrations included the anions, Cl, NO_3_, and SO_4_, paired in every combination with the cations, Mg, K, and Na. Certain salts of NH_4_, borate, Ca, Cs, ClO_3_, Fe, ClO_4_, and PO_4_ also were examined. Strong growth was observed among the isolates in Salt Plains media supplemented with 2 M MgCl_2_, 1 M Mg(NO_3_)_2_, 2 M MgSO_4_, 0.6 M K chlorate, 4 M KCl, 3 M KNO_3_, 0.8 M K_2_SO_4_, 3 M NaCl, 2 M NaNO_3_, 2 M NaH_2_PO_4_, and 1.5 M Na_2_SO_4_, often with modest growth at higher concentrations. Logistic growth curves were fitted to estimate the rate of population increase (*r*) and carrying capacity (*K*) for each replicate. Effects on bacterial growth were best explained by statistical models that included anion, cation, water activity and all their 2-way and 3-way interactions. Salt-specific effects suggest that the concentration of any single ion is not consistently the reason behind the inhibition of microbial growth. Knowledge of the tolerances of terrestrial microbes to salts at extremely high concentrations sets limits for the permissible conditions of extraterrestrial habitable regions and for the risks of forward planetary contamination.

**Supplementary Information:**

The online version contains supplementary material available at 10.1007/s00203-025-04418-9.

## Introduction

Liquid water on Mars appears to be rare near the surface due to its aridity and low temperature. Salts can depress the freezing point of water, hence remaining liquid at lower temperature. The chlorate salts of Mg and Na have freezing points of − 69 and − 23 °C, respectively (Hanley et al. 2012). Eutectic concentrations of (per)chlorate salts have yet to be detected on Mars, but these salts were present at ~ 0.6% near the Phoenix lander (Hecht et al. 2009; Kounaves et al. 2010; Ming et al. 2014; Clark and Kounaves 2016). However, there appears to be sufficient moisture and warmth at the Curiosity landing site to create eutectic perchlorate brines during discrete periods and the potential persistence of Fe sulfate brines has been suggested (Chevrier and Altheide 2008; Chevrier et al. 2009; National 2023; Hu et al. 2016; Jänchen et al. 2016; Primm et al. 2017; Nair and Unnikrishnan et al. 2020; Rivera-Valentín et al. 2020). It has been proposed that a lake buried under the polar ice cap of Mars is a (per)chlorate brine (Orosei 2018). Martian surface temperatures are currently too low for liquid brines of NaCl and MgSO_4_, which have eutectic temperatures of − 21 and − 4 °C, respectively (Toner et al. 2014). In environments further below the surface, warmer temperatures may allow for these brines to form. In fact, the most likely habitable environments on Mars are expected to be salty, including brine veins in ices, cave efflorescences, and salt evaporite minerals (Carrier et al. 2020).

The chemical conditions in the oceans and icy crusts of ocean worlds have not been adequately determined. Proposed salinities for the oceans of Enceladus, Europa, and Ganymede range from approximately that of the ocean on Earth (~ 3% NaCl) to various concentrations of MgSO_4_, and as high as nearly saturated with Na_2_SO_4_, MgSO_4_, and NaCl (Chyba and Phillips 2001; Hand et al. 2007; Brown and Hand 2013; Vance et al. 2014; Fifer et al. 2022; Cockell et al. 2024; Neveu et al. 2024). Diapirs and sills, pockets of liquid brines in the ice crusts of ocean worlds, may concentrate salts, creating high physical pressure leading to geysers or cryovolcanic seeps (Postberg et al. 2011; Kattenhorn and Prockter 2014; Vorburger and Wurz 2021; Culberg et al. 2022; Lesage et al. 2022; MacKenzie et al. 2022). The ejected material forms sublimation lag on the surface, which could be salty, and this may later subsume back into the subsurface (National 2023; Cockell et al. 2024). Ammonia-water solutions can depress the freezing point to −100 °C, implicating these in the oceans of Pluto, Titan, and Kuiper Belt Objects (Brown and Hand 2013; Nimmo and Pappalardo 2016).

Environments with high solute concentration pose unique challenges to microbial survival and proliferation (Grant 2004; Schneegurt 2012). Certain qualities of solutes are clearly deleterious to microbes, even at modest concentrations. Heavy metals such as Hg directly inhibit enzymes and thereby block cellular functions. Detergents and organic solvents, including ethanol, are broadly disinfectant by disrupting cellular membranes and denaturing proteins. Many solutes, including salts and sugars, are not acutely toxic, but at high concentration, create chemical and physical conditions only tolerated by specialized guilds of microbes. High solute concentration limits the habitability of environments for terrestrial microbes on Earth, Mars, or ocean worlds, as well as potential native life beyond Earth. If terrestrial microbes carried by spacecraft can survive and/or proliferate on another world, these may negatively impact forward planetary protection, specifically, interfering the search for faint signs of life, past or present, and with possible native ecosystems. Hence, extremophilic microbes are often examined as model organisms in astrobiology studies (Hallsworth et al. 2021; Emlik and Marakli 2023; Schultz et al. 2023; dos Santos et al. 2024).

Certain characteristics of brines and syrups are clearly inhibitory to common microorganisms. Solutions with high solute concentration exert greater osmotic pressure on cells. For most microbes, this results in loss of water from the cell, shriveling, and death. Osmotolerant microbes often respond to solutions with high solute concentration by filling their cytoplasm with solutes that are compatible with cellular process, to balance the osmotic pressure of the bathing medium (Grant 2004). Betaine, ectoine, and glycerol are common compatible solutes used by salinotolerant bacteria. Solutions with high solute concentration also exhibit a related quality, their water activity (*a*_*w*_) is substantially lower than that of pure water. A low *a*_*w*_ is clearly associated with the inhibition of microbial growth and survival, since it limits the availability of water in a solution for cellular uptake and use (Beuchat 1981; Harris 1981; Grant 2004; Rummel et al. 2014; Stevenson et al. 2015, 2017; Hallsworth 2019). Thus, habitability is expected to be lower in regions with low *a*_*w*_.

The particular physical qualities of ionic solutes may further influence microbial growth. The ionic strength of a solution is dependent on solute concentration and ion charge, that is why ionic strength is higher in brines of divalent ions, such as those in epsomic environments, as suggested for Mars and the ocean worlds (Brown and Hand 2013; Carrier et al. 2020). Ionic strength can render environments uninhabitable despite the presence of bioavailable water (Fox-Powell et al. 2016). A related feature of ions, their charge density, takes ionic radii (hydrated and unhydrated) into account and may influence the deleterious interactions of brines with cells (Waajen et al. 2020). Chaotropicity and kosmotropicity, the destabilization and stabilization of macromolecular complexes, respectively, lead to Hofmeister (1888) effects such as the aggregation or dispersal of macromolecules (Ball and Hallsworth 2012). These characteristics of ions and salts may have an influence on microbial growth and survival. Microbes respond in complex ways, based on their genetics, to each quality of their environment. By examining the growth of diverse bacteria in various salts at a wide range of concentrations, trends may emerge that explain cellular responses to individual ions, their salts, or colligative properties of the brines. We thereby may connect certain parameters of solutes and their solutions to cellular growth responses and suggest their role in habitability. Each ion and salt is expected to exert specific effects on cells based on its physical qualities and its interactions with counterions (Scott 1953; Chirife 1994; Cray et al. 2012).

Natural environments with high solute concentration on Earth are relatively limited in chemical diversity. Most hypersaline environments are rich in halite, while environments rich in epsomite, mirabilite, and potash are less prevalent. There typically aren’t natural brines of nitrates, chlorates, and perchlorates, or brines of cations such as Li or NH_4_. Syrups are typically limited to honey, sugar cane bagasse, aphid droplets, drying sap, and flower nectar. Since microbes are expected to be adapted to their natural environments, finding tolerances to uncommon or unnatural brines would seem to be fortuitous. However, microbes from common oligohaline soils show remarkable tolerance to high salinity and sugar concentrations that greatly lower *a*_*w*_ and high tolerance to (per)chlorate brines has been reported (Nielsen et al. 1995; Echigo et al. 2005; Al Soudi et al. 2017; Fredsgaard et al. 2017b; Howell et al. 2022).

The current report examines the effects of ions and salts on the growth of 18 aerobic salinotolerant bacteria from the Gram-negative genus *Halomonas* and from the Gram-positive genera *Bacillus*,* Marinococcus*,* Nesterenkonia*,* Planococcus*,* Salibacillus*, and *Terribacillus* (Caton et al. 2004; Kilmer et al. 2014). An iterative matrix of ions and their resulting salts included the anions chlorate, chloride, nitrate, perchlorate, phosphate, and sulfate, coupled with the cations NH_4_, Ca, Mg, K, and Na. Time series of bacterial growth were generated in media supplemented with increasing concentrations of each salt, until growth ceased or the medium was saturated with salt. Growth curves were fitted to each time series to generate estimates of the growth rate (*r*) and carrying capacity (*K*). Competing models then were compared in an iterative fashion, using cation and anion identity, salt-specific combinations, and physical parameters of the ions and brines (ionic strength, molarity, and *a*_*w*_, among others), to identify which set of parameters best explained observed effects on bacterial growth. Determining the limits of life at high salinity and the physical parameters leading to the observed cellular responses in laboratory analog systems, sets limits for the potential habitability of locales with high salinity on Mars and the ocean worlds.

## Materials and methods

### Organisms

A group of 18 salinotolerant aerobic bacterial isolates within existing collections from Hot Lake, WA (saturated with epsomite; Kilmer et al. 2014) and the Great Salt Plains, OK (saturated with NaCl; Caton et al. 2004) was chosen for this study based on salinotolerance and taxonomic spread (Table 1). Cultures were maintained at room temperature on agar slants of Salt Plains (SP) medium (containing per liter: NaCl, 1 g; KCl, 2.0 g; MgSO_4_·7H_2_O, 1.0 g; CaCl_2_·2H_2_O, 0.36 g; NaHCO_3_, 0.06 g; NaBr, 0.23 g; FeCl_3_·6H_2_O, 1.0 mg; trace minerals, 0.5 ml; tryptone, 5.0 g; yeast extract, 10.0 g; glucose, 1.0 g; final pH 7.0) supplemented to 10% NaCl (Caton et al. 2004).

**Table 1:** Bacterial isolates used in the current study

Strain	Identification	GenBank Accession
GSP3	*Halomonas venusta*	AY505527
GSP10	*Bacillus megaterium*	AY505510
GSP11	*Terribacillus halophilus*	AY553069
GSP17	*Salibacillus marismortui*	AY505533
GSP21	*Halomonas salina*	AY553072
GSP63	*Bacillus licheniformis*	AY553106
HL11	*Marinococcus* * halophilus*	KC705247
HL12	*Halomonas* * venusta*	KC705248
HL14	*Halomonas* * venusta*	KC705250
HL20	*Planococcus* * pelagicus*	KC705257
HL54	*Marinococcus* * halophilus*	KC705293
HL55	*Bacillus* * licheniformis*	KC705294
HL64	*Nesterenkonia* * halotolerans*	KC705304
HL68	*Bacillus* * licheniformis*	KC705294
HL76	*Nesterenkonia* * halotolerans*	KC705317
HL80	*Planococcus* * salinarum*	KC705322
HL82	*Halomonas* * venusta*	KC705324
HL91	*Planococcus* * salinarum*	KC705334

GSP and HL strains were isolated from the Great Salt Plains and Hot Lake, respectively

### Growth measurements for salinity tolerance

Bacterial cultures were grown in SP medium supplemented with various salts at a range of concentrations, without adjusting pH. Shake-tubes (2 ml in capped 13 × 100 mm tubes) were lightly loop-inoculated (below 0.05 OD units) from agar slants and incubated at room temperature on a rotary shaker (150 rpm; 1-in stroke dia). Culture density was determined by absorbance spectroscopy at 600 nm (ThermoFisher Genesys 10S) at 2-d intervals for 12 d after inoculation, using uninoculated blanks of the corresponding medium as the background. Triplicate shake-tubes were used for each growth curve and mean values are reported with SD.

### Model development

Values for the physical parameters of the brines, salts, and ions included in the models used to determine effects on microbial growth are given in Table S1. The *a*_*w*_ of each medium was measured using an AquaLab Series 3 *a*_*w*_ meter (Decagon Devices, Inc., Pullman, WA). The instrument was calibrated with standard NaCl solutions and run at room temperature. Ionic strength and percent saturation were calculated (IUPAC 1987). Alpha, hydrated radii, hydration bond length, ionic radii, molality, osmolarity, osmotic coefficients, osmotic pressure, and van t’ Hoff factors were taken or calculated from previous literature (Kielland 1937; Nightingale et al. 1959; Hamer and Wu 1972; IUPAC 1987; Volkov et al. 1997; Moin et al. 2010; Mondal et al. 2014).

**Fig. 1:**
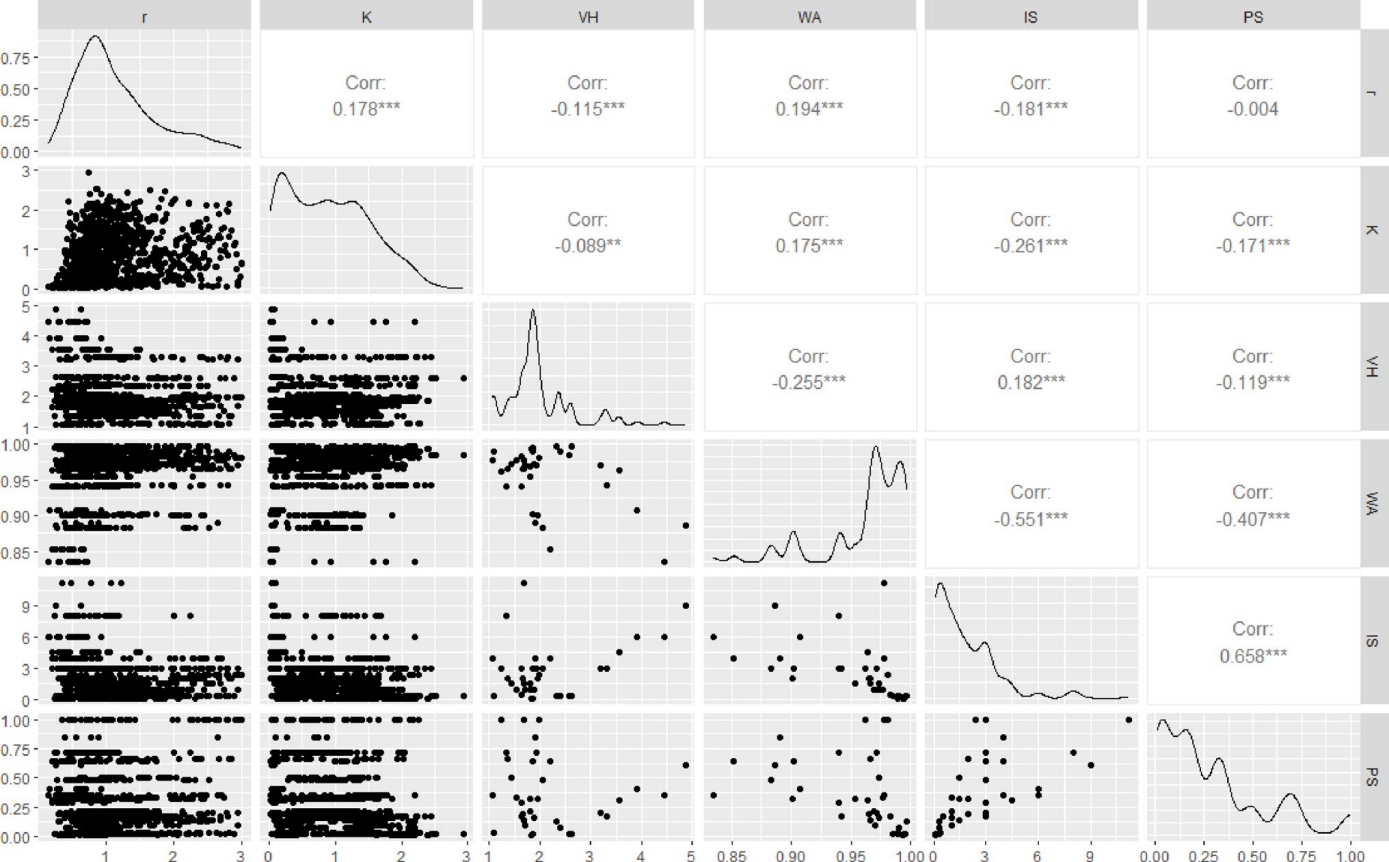
Assessment of ion parameters for correlation along fitted parameters growth rate (*r*) and carrying capacity (*K*) to identify multiple collinear predictor variables. The four ion parameters used for subsequent models were ionic strength (IS), percent saturation (PS), van t’ Hoff factor (VH), and *a*_*w*_ (WA)

To avoid multiple collinear predictor variables, ion parameters were assessed for correlation along with the fitted parameters *r* and *K*. The four ion parameters used in subsequent models, namely, ionic strength, percent saturation, van t’ Hoff factor, and *a*_*w*_, were selected based on their lack of correlation to each other (correlation coefficient under 0.70), continuous distributions of values across salt concentrations (i.e., behave more like continuous than categorical variables), and previous studies on growth effects observed with salinity, particularly *a*_*w*_ and ionic strength (Fig. 1). Molality, molarity, osmolarity, osmotic coefficient, and osmotic pressure were subsequently omitted from model comparisons as they were correlated with the four chosen ionic parameters and had poorer predictive power in preliminary models. Alpha, charge, hydrated radii, rH hydrated, rs Stokes, rx crystal, and the cation and anion oxygen bond lengths of the salts did not vary sufficiently between ions and salts to be significant factors in the analysis.

Logistic growth curves were fitted with the curve-fitting tool in MATLAB (2018) to estimate rate of population increase (*r*) and carrying capacity (*K*) for each individual culture replicate. Goodness of fit (*R*^2^) for logistic growth curves, maximum and minimum OD readings, and the last OD reading in each series were recorded along with fitted parameters (*r*, *K*) for post-model assessment. Curves were fit iteratively by recording goodness of fit into a matrix with identifying information (e.g., bacterial strain, replicate, salt, salinity) and then curve-fit data was included in the statistical analyses, if it met the following criteria: *R*^*2*^ values > 0.5, *r* value between 0 and 3, *K* > 0, and values of *K* that were no more than twice the final OD reading.

### Statistical analyses

To determine whether effects on bacterial growth were salt-specific, initial analyses used a subset of “Core Ions” for which growth curves were obtained across a fully factorial combination of three anions (chloride, nitrate, sulfate) and three cations (magnesium, potassium, sodium). Core Ions were used to directly test whether salt-specific effects (anion by cation interactions), anion identity, or cation identity best explained variation in growth curve parameters. Strain ID was included as a random effect in all models. Fourteen a priori candidate models (generalized linear mixed-effects models) were built to compare the relative ability of anion, cation, ionic parameters, and salt-specific effects (anion by cation interaction term) to explain effects on bacterial growth. Analyses were run in the R statistical environment with mixed models created through the ‘lme4’ package and model comparisons run through the ‘bbmle’ package (Bates et al. 2015; Bolker and R Development Core Team 2022; R Core Team 2022). Candidate models included all four combinations of anion and cation plus an ionic parameter and all of their interactions (2-way and 3-way), all four combinations of models with anion and cation, their interaction and an ionic parameter, all four models with only an ionic parameter, a model with only anion, cation and their interaction, a model with anion and cation without an interaction effect, and an intercept model.

Data from the broader set of “All Ions” (additionally including the anions ClO_3_, and PO_4_ and the cations NH_4_ and Ca) examined for their effects on bacterial growth parameters did not include fully factorial combinations of the ions, given that certain salts were either sparingly soluble or not commercially available. Since the interaction term between anion and cation was in the top model for the Core Ions, the models built for the entire data set of All Ions used salt identity in lieu of anion, cation, or their interaction. The remaining parameters for the All Ions dataset were determined by the top model in the Core Ions analyses.

## Results

### Bacterial growth in brines

**Fig. 2:**
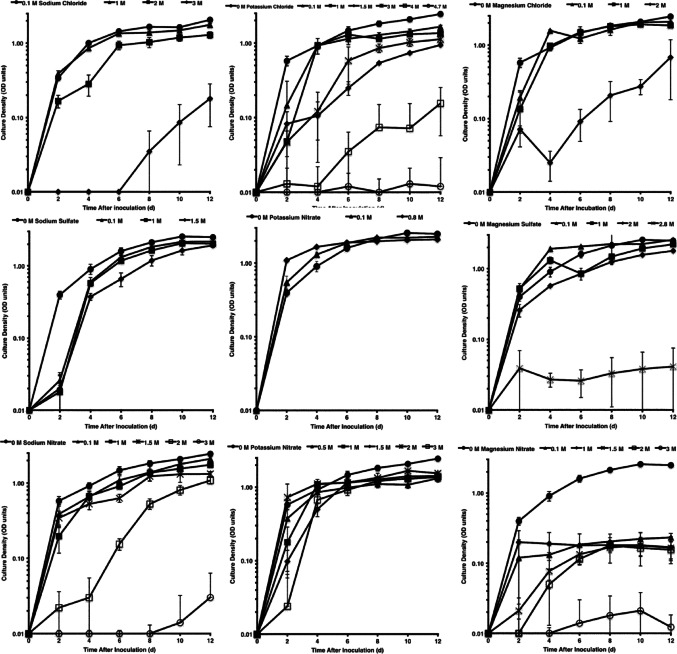
Representative bacterial logistic growth curves based on culture density for the 9 salts comprising the Core Ions at various concentrations. Growth curves with *Halomonas venusta* str. HL12 are presented for MgCl_2_, KCl, KNO_3_, NaCl, and NaNO_3_. Growth curves with *Marinococcus halophilus* str. HL11 are presented for Mg(NO_3_)_2_, MgSO_4_, K_2_SO_4_, and Na_2_SO_4_

Salt Plains medium was supplemented with various salts at a range of concentrations to measure their effects on the growth of 18 salinotolerant bacterial isolates. Over 4000 logistic growth curves were obtained for analysis. Representative growth curves for *Marinococcus* sp. str. HL11 or *Halomonas* sp. str. HL12 are shown for each of 9 salts that comprise the Core Ions, an iterative matrix of the cations Mg, K, and Na coupled with the anions Cl, NO_3_, and SO_4_ (Fig. 2). Growth was strong across a range of molar concentrations for all of these salts, with the exception of Mg(NO_3_)_2_. Measurable increases in culture density were slowed or delayed at the highest concentrations tested for most of the salts examined (Table 2). For MgSO_4_, KCl, KNO_3_, K_2_SO_4_, and Na_2_SO_4_, the highest concentrations tested are near or at saturation. Cursory examination of this data suggests that growth tolerances were greater at higher degrees of saturation for the salts containing potassium or sulfate. Statistical models were generated to determine correlations between growth rates (*r*) or maximum culture densities (*K*), the suite of ions and salts examined, and their physical characteristics.

**Table 2:** Maximum growth tolerances observed among the 18 salinotolerant bacteria in various salts, with strong and modest growth exhibiting culture densities of >1.0 and >0.25 OD units at 600 nm, respectively

Salt	Limit (M)	Strong Growth	Modest Growth
NH_4_Cl	1.0	HL12, HL64, HL68, HL91	HL55
(NH_4_)_2_SO_4_	1.0	HL12, HL20, HL64, HL91	HL55, HL68, HL80, HL82, GSP10, GSP11, GSP21, GSP63
CsCl	0.5	HL12, GSP21	
MgCl_2_	3.0		HL91
Mg(NO_3_)_2_	2.0		HL11, HL54,
Mg(ClO_4_)_2_	0.1	HL11, HL12, HL20, HL54, HL55, HL64, HL68. HL15M, GSP3, GSP10, GSP11, GSP17, GSP21, GSP63	HL82
MgSO_4_	2.8		GSP3
KClO_3_	0.5	All	
KCl	4.0	HL91	HL12, HL64
KNO_3_	3.0	HL11, HL12, GSP63	HL64, HL68, HL82, HL91
K_2_SO_4_	0.8	All except three	HL55, GSP3, GSP11
Na_2_B_4_O_7_	0.5		HL12, HL55, GSP17, GSP21
NaCl	4.0		HL12
NaNO_3_	3.0		HL91
Na_2_HPO_4_	3.0		All
Na_2_SO_4_	1.5	HL11, HL12, HL14, HL20, HL64, HL68, HL76, HL91, GSP21	HL1M, GSP3, GSP10, GSP11, GSP17, GSP63

Beyond the Core Ions, the All Ions set included salts containing NH_4_, borate, Ca, Cs, ClO_3_, Fe, ClO_4_, and PO_4_. Not all combinations of ions were commercially available or appreciably soluble in water, and some were clearly toxic at low concentrations that did not affect *a*_*w*_. Phosphate was coupled with K and Na, but Mg and Ca phosphates are insoluble in water. Growth was observed at 2 M, but not 3 M NaH_2_PO_4_, while K_2_HPO_4_ showed growth at 1 M but not 2 M. Our previous study with (per)chlorate salts demonstrated growth in media containing 1 M perchlorate salts and nearly 3 M chlorate salts (Al Soudi et al. 2017). The bacterial isolates were tolerant to both (NH_4_)_2_SO_4_ and NH_4_Cl at 1 M but not at 2 M concentrations. Calcium salts seemed more inhibitory overall, with tolerance observed only to 0.5 M. Salts of borate, Cs, and Fe were not included in the model, with both CsCl and Na_2_B_4_O_7_ permitting growth at 0.5 M but not at 1 M. Fe salts appeared more toxic with ~ 0.1 M preventing growth, as previously observed for transition metals (Marnocha et al. 2011; Amils 2016; Fox-Powell and Cockell 2018).

### Effects of salts and ionic properties on bacterial growth

**Fig. 3:**
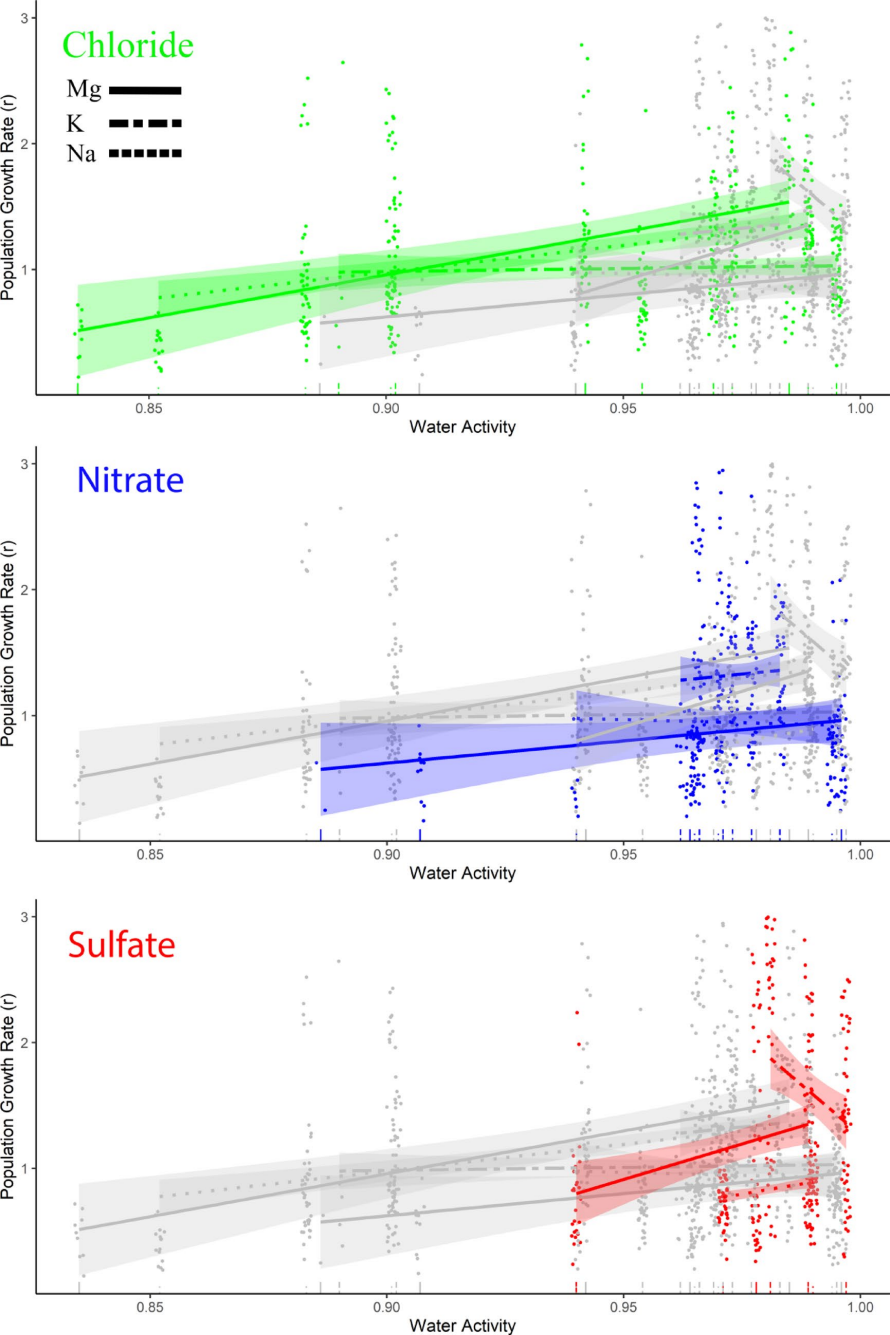
Effect of *a*_*w*_ on population growth rate (*r*) of 18 salinotolerant bacteria for the 9 salts comprising the Core Ions as fitted by Generalized Linear Mixed Models. Separate panels highlight changes in *r* across *a*_*w*_ for each anion

**Fig. 4:**
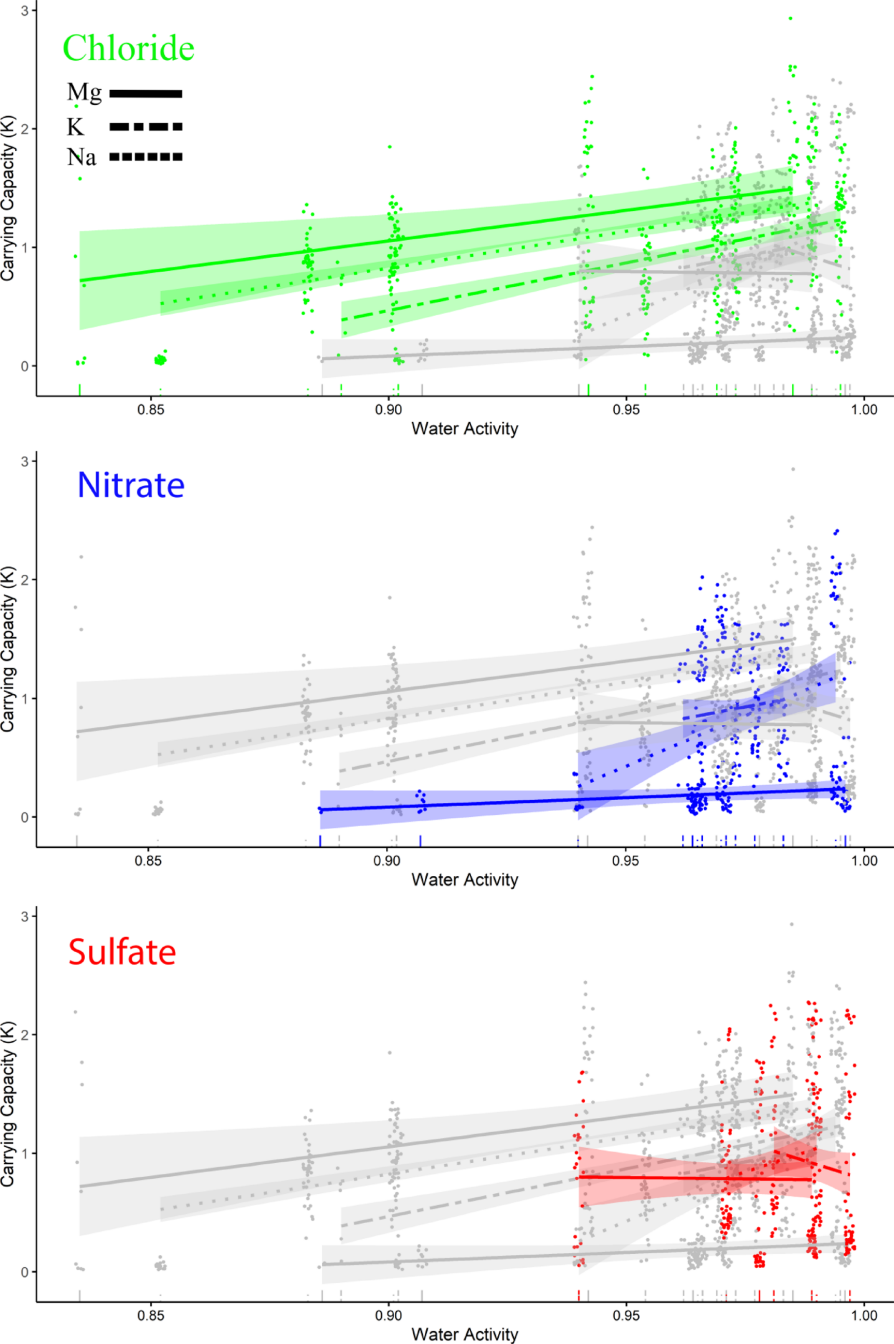
Effect of *a*_*w*_ on carrying capacity (*K*) of 18 salinotolerant bacteria for the 9 salts comprising the Core Ions as fitted by Generalized Linear Mixed Models. Separate panels highlight changes in *K* across *a*_*w*_ for each anion

Bacterial growth rates (*r*) for the 9 salts of the Core Ions (1,138 curves) were best explained by models that included anion, cation, *a*_*w*_, and all their 2-way and 3-way interactions (Tables 3 and 4). Candidate models including the intercept (null model), anion and cation without interaction, one-factor models of the colligative properties (ionic strength, percent saturation, van t’ Hoff factor, and *a*_*w*_), or three-factor models that included anion and cation (with their interaction), and an additive effect of a colligative property were less supported by a wide margin. The observation of salt-specific effects suggests that the concentration of any single ion is not consistently the reason behind the inhibition of microbial growth. Bacterial growth rates generally increased as *a*_*w*_ approached 1, the *a*_*w*_ of pure water, but the rates of increase were salt-specific (χ^2^_4,1138_ = 29.9, *p* < 0.0001) (Fig. 3). The only salt to show a negative relationship between *r* and *a*_*w*_ was K_2_SO_4_, but it also was the salt with the smallest range of *a*_*w*_ values. This may reflect localized optima with a slightly lower *a*_*w*_ for this salt. The Core Ions exhibited effects on carrying capacity (*K*) that were similar to those on *r*, where the results were best explained by models that included anion, cation, *a*_*w*_, and all their 2-way and 3-way interactions (Tables 3 and 4). Again, maximum culture density, the carrying capacity, generally increased with *a*_*w*_ as it approached 1 (χ^2^_4,1138_ = 45.0, *p* < 0.0001) (Fig. 4).

**Table 3:** Analysis of deviance for models of the effects of ion parameters on the growth responses *r* and *K* for Core Ions and All Ions, using Type III Wald chi-square tests

	Chisq	df	*P*
**Effects on ** ***r*** ** for All Ions**			
Anion	7.35	2	0.025*
Cation	6.16	2	0.046*
*a*_*w*_	27.71	1	<0.001***
Anion:Cation	30.94	4	<0.001***
Anion:*a*_*w*_	7.47	2	0.024*
Cation:*a*_*w*_	7.19	2	0.027*
Anion:Cation:*a*_*w*_	29.90	4	<0.001***
**Effects on ** ***K*** ** for All Ions**			
Anion	6.02	2	0.049*
Cation	14.18	2	0.001***
*a*_*w*_	32.54	1	<0.001***
Anion:Cation	42.97	4	<0.001***
Anion:*a*_*w*_	9.15	2	0.010*
Cation:*a*_*w*_	12.21	2	0.002**
Anion:Cation:*a*_*w*_	44.98	4	<0.001***
**Effects on ** ***r*** ** for All Ions**			
Salt	71.08	13	<0.001***
*a*_*w*_	0.27	1	0.603
Salt:*a*_*w*_	71.13	13	<0.001***
**Effects on *K*** ** for All Ions**			
Salt	216.61	13	<0.001***
*a*_*w*_	13.93	1	<0.001***
Salt:*a*_w_	217.01	13	<0.001***

**Table 4:** Correlations of Type III ANOVA models of the effects of brine parameters on the growth responses *r* and *K* for Core Ions

Model^a^	dAIC_c_	df	Weight
**Effects on ** ***r*** ** for Core Ions**			
Anion * Cation * *a*_*w*_	0.0	20	1
All remaining models	>33.3		<0.001
Intercept	249.8	3	<0.001
**Effects on ** ***K*** ** for Core Ions**			
Anion * Cation * *a*_*w*_	0.0	20	0.943
Anion * Cation * DS	5.6	20	0.057
All remaining models	>39.1		<0.001
Intercept	455.1	3	<0.001

DS, degree of saturation; *a*_*w*_, water activity

**Fig. 5:**
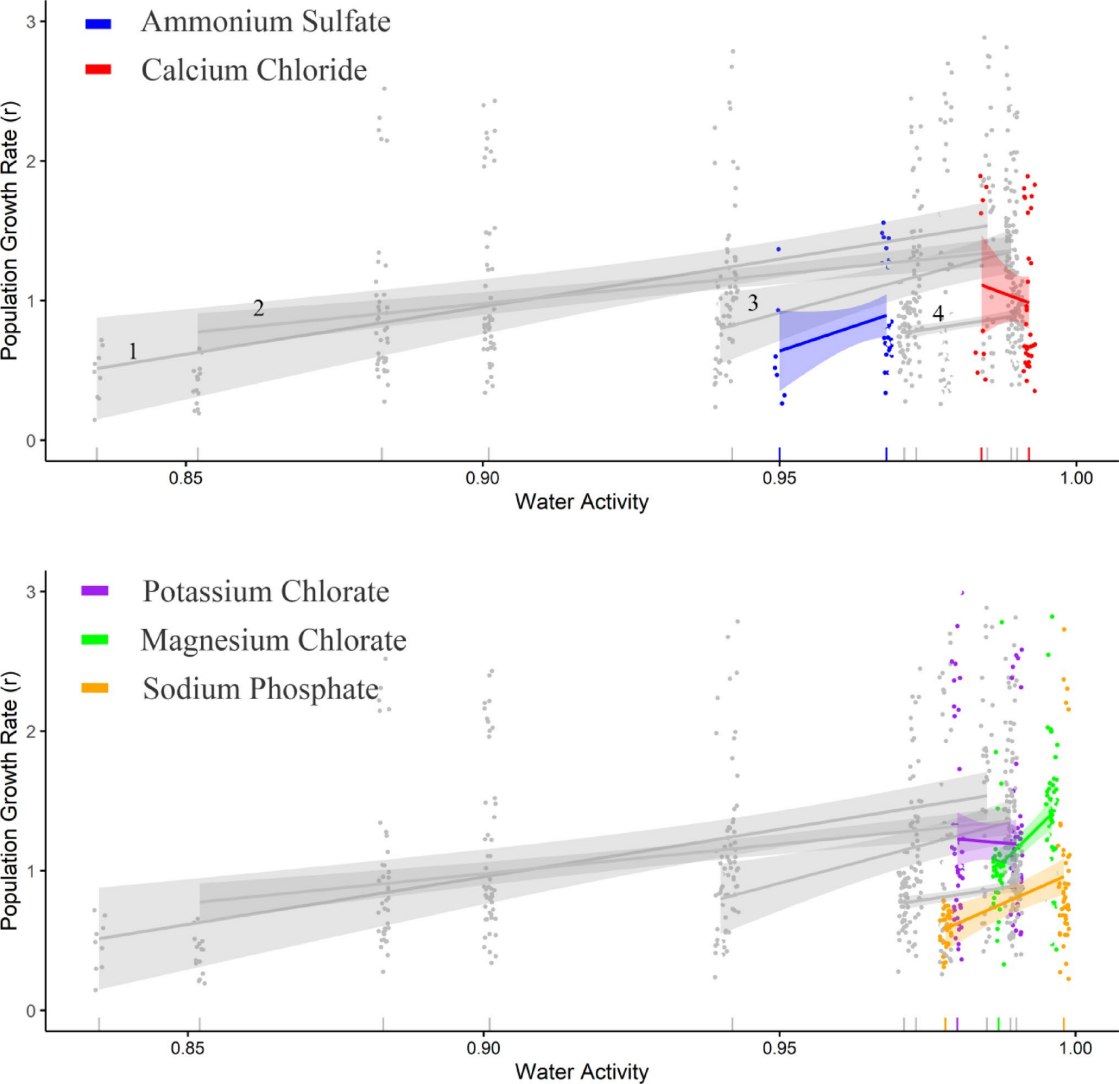
Effect of *a*_*w*_ on population growth rate (*r*) of 18 salinotolerant bacteria for the salts comprising the All Ions set as fitted by Generalized Linear Mixed Models. Separate panels highlight changes in *r* for anions and cations beyond the Core Ions. Grey reference curves depict (1) MgCl_2_, (2) NaCl, (3) MgSO_4_, and (4) Na_2_SO_4_

**Fig. 6:**
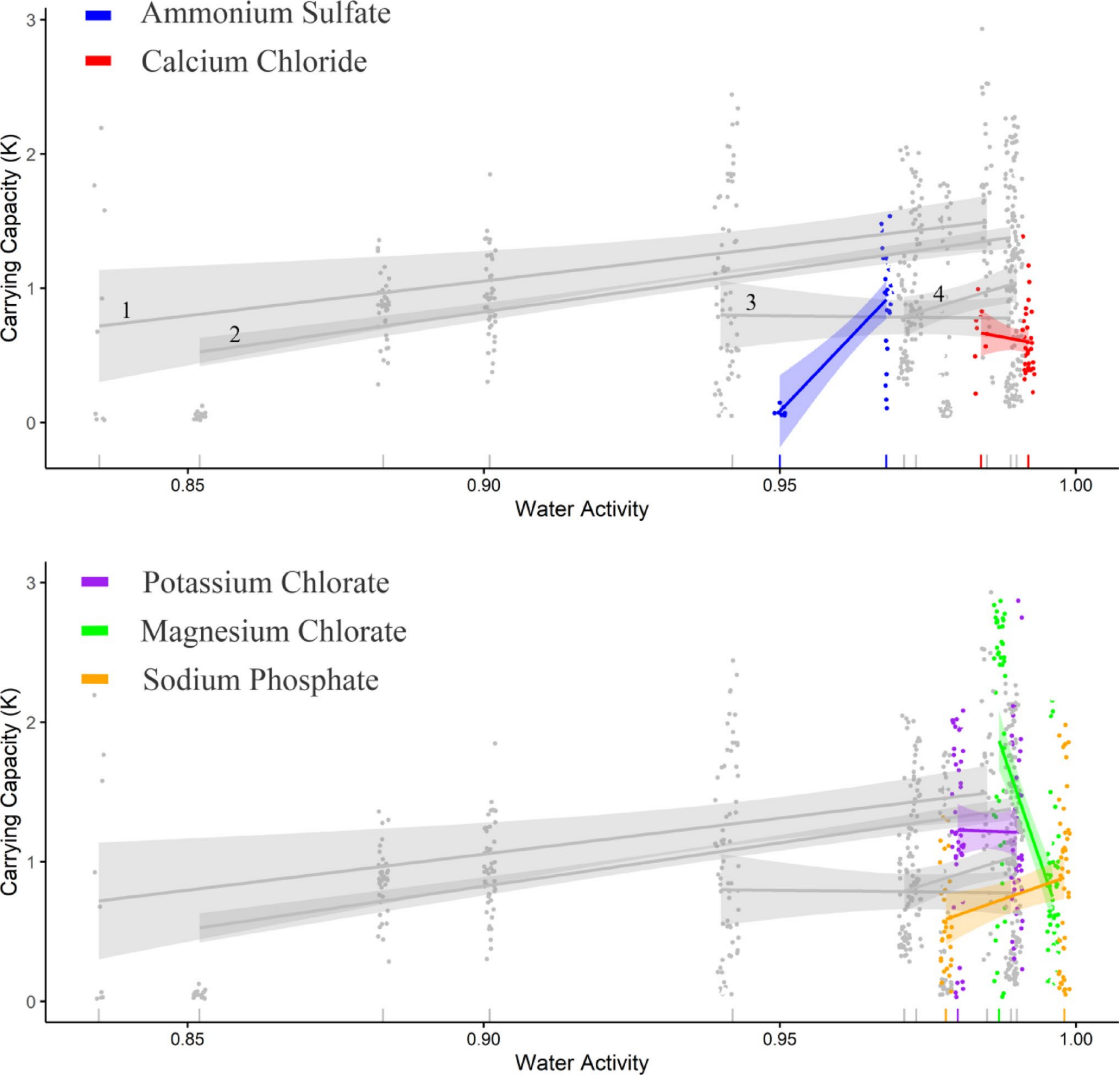
Effect of *a*_*w*_ on the carrying capacity (*K*) of 18 salinotolerant bacteria for the salts comprising the All Ions set as fitted by Generalized Linear Mixed Models. Separate panels highlight changes in *K* for anions and cations beyond the Core Ions. Grey reference curves depict (1) MgCl_2_, (2) NaCl, (3) MgSO_4_, and (4) Na_2_SO_4_

Bacterial *r* and *K* for the All Ions set of salts (1,538 curves) showed strong salt-specific effects across gradients of *a*_*w*_. As with the Core Ions, the dataset of All Ions showed generally consistent salt-specific increases in both *r* and *K* as *a*_*w*_ approached 1 (Figs. 5 and 6). The ranges of concentration tested and their *a*_*w*_s were relatively small for these ions, due to their limited solubility or substantial toxicity.

## Discussion

A diverse group of salinotolerant bacteria exhibited substantial growth tolerance to a variety of salts at high concentration. Tolerances were observed at near saturation for several salts (MgSO_4_, KCl, KNO_3_, K_2_SO_4_, and Na_2_SO_4_). It is well known that natural haline environments harbor a wealth of microbes despite extreme salinities, even to saturation (Grant 2004; McGenity and Oren 2012). While less frequently studied, epsomic environments with high concentrations of divalent ions also appear to maintain rich microbial communities (Crisler et al. 2012; Kilmer et al. 2014; Pontefract et al. 2017). Our results and those of Onishi et al. 1980 showing extreme microbial growth tolerance to K salts (to 4 M), suggest that potash environments might have rich microbial communities as well. There are limited reports on the microbial ecology of natural potash (Norton et al. 1993; Naumovich et al. 2015; Payler et al. 2019). Nitrate salts were compatible with our salinotolerant isolates, but not to concentrations above 1 M or percents saturation as high as chloride and sulfate salts. Kamekura and Onishi (1982) reported growth of a *Micrococcus* at 2 M KNO_3_ and 3.5 M NaNO_3_. Natural environments with molar concentrations of nitrates or (per)chlorates are not readily found on Earth, however, the latter may be important on Mars (Kounaves et al. 2010; Jackson et al. 2015). We previously have demonstrated strong tolerance to chlorate salts (to 2.7 M), which are better tolerated than corresponding perchlorate salts (to 1 M) (Al Soudi et al. 2017).

The present study observed greater growth inhibition with increasing concentration across a range of salts. Working towards an explanation for these observations, we tested a matrix of salts to identify brine parameters that lead to reductions of intrinsic growth rate or maximum culture density for a taxonomically diverse group of bacteria. Mixed effects models demonstrated that the ionic species constituting the brines have a substantial effect on both *r* and *K*. Certain ions appeared more inhibitory to growth than others, such as Ca, Fe, and ClO_4_ (Table 2). However, specific solute effects (Scott 1953) for the salts were important contributors to the observed effects on bacterial growth. For instance, Mg(NO_3_)_2_ was more inhibitory than MgCl_2_ or MgSO_4_, while also being more inhibitory than KNO_3_, at the same concentration (Fig. 2). Magnesium chloride was more inhibitory than MgSO_4_, KCl, or NaCl at the same concentration. Effects on growth that are specific to individual salts previously have been proposed to explain the interactions between brines and microbial growth (Scott 1953; Chirife 1994; Cray et al. 2012; Fredsgaard et al. 2017a; Carte et al. 2024).

Low *a*_*w*_ is considered by many to be the key physical quality of brines that most strongly effects microbial growth rates (Rummel et al. 2014; Hallsworth 2019; Carrier et al. 2020). We confirm this observation by finding a strong correlation between the growth parameters *r* or *K* with *a*_*w*_, even for the extremely salinotolerant microbes tested. The effects of *a*_*w*_ on growth were salt-specific for the Core Ions (three-way interaction between anion, cation, and *a*_*w*_). Ionic strength previously was suggested as a possible determining feature of the effects of brines on microbial growth (Fox-Powell et al. 2016). The extension is that environments rich in divalent ions, such as the near-surface of Mars, would be particularly inhibitory. Our models show that *a*_*w*_ was best at explaining salt-specific effects on bacterial growth rates by a wide margin. However, the permissible brines in the current report had modest ionic strength, well below the inhibitory levels reported previously. The current study did not measure the chaotropicity of the media, but this would be expected to follow the trends for Hofmeister effects (Hofmeister 1888; Cray et al. 2012). Chaotropicities were not available for many of the salts tested in the current study and therefore could not be included in the model. However, a comparison of reported chaotropicities (Cray et al. 2012) of certain salts used here to observed growth tolerances do not appear to be highly correlated. While MgCl_2_ is strongly chaotropic, weak growth was observed for HL91 at 3 M, although MgCl_2_ was more inhibitory than the kosmotropic salts MgSO_4_, KCl, or NaCl at the same concentrations. The mildly chaotropic salt NH_4_Cl was tolerated to 1 M, the same maximum observed for the strongly kosmotropic salt (NH_4_)_2_SO_4_.

Heavy brine offers advantages for the survival of microbes in extremely dry, salty, and cold environments similar to Mars (Rummel et al. 2014; Carrier et al. 2020). High solute concentration lowers the freezing point of water, providing liquid water in cold places, thereby enhancing their habitability, and spatially expanding Special Regions where life is most plausible. In extreme environments, liquid water may only exist at the eutectic conditions for a salt or in thin layers on salt crystals (Jabosky et al. 2004; Hansen-Goos and Wettlaufer 2010; Bruzewicz et al. 2011; Hanley et al. 2012; Hansen-Goos et al. 2014; Toner et al. 2014). In addition, brines are chemically stable environments that evaporate slowly, temporally extending their persistence in cold environments. As salty water freezes, ice phases of pure water form, creating brine veins of increasing salinity and creating briny inclusions that retain liquid water, which can maintain microbes (Litchfield 1998; Junge et al. 2004, 2006; Mader et al. 2006; Ohno et al. 2014).

Salt evaporite minerals formed in hyperarid environments can provide a refuge for microbes that become entrapped in briny fluid inclusions within salt crystals. Microbes can survive for extended periods in these sequestered saturated brines, which may represent the last habitable water in an aridifying environment (Rothschild et al. 1994; Vreeland et al. 1998; McGenity et al. 2000; Schubert et al. 2009, 2010; Stan-Lotter and Fendrihan 2015; Elabed et al. 2019; Huby et al. 2020). Salt crystals containing microbes can be transported great distances by winds, potentially dispersing entrapped microbes to more favorable locations (Wheeler 1985; Dieckmann et al. 1999; Mayol et al. 2017). As moisture returns to an arid environment, or if the crystals deposit in a moister location, sufficient humidity will cause the salt evaporites to deliquesce into brines, providing a liquid phase for microbes that were entrapped in salt crystals. We previously have demonstrated bacterial survival and growth in eutectic brines and in deliquescent liquids (Wilks et al. 2019; Cesur et al. 2022). Thus, fluid inclusions in salt crystals may represent both the last habitable water during aridification and the first liquid water formed when more moisture is available.

These considerations are particularly relevant for Mars, a cold hyperarid environment. All the wet environments on Mars proposed for investigation at the Carlsbad Mars Extant Life Conference, namely ices, subsurface, caves, and evaporites, involve salt brines, efflorescences, precipitates, and evaporite minerals in a way that extends the range of their potential habitability (Carrier et al. 2020). Mars is not only colder and drier than Earth, but also far richer in divalent ions of sulfate, often paired with Mg and Ca (Clark 1993; Wänke et al. 2001; Clark et al. 2005). The current study reports high tolerances for sulfate salts, with MgSO_4_ tolerance seen at saturation. In addition, Mars is dry enough to support the widespread presence of (per)chlorate salts, which are rare on Earth (Rummel et al. 2014; Fischer et al. 2016; Primm et al. 2017; Pál and Kereszturi 2020). We previously have shown substantial tolerance to chlorate salts (to 2.7 M), although perchlorate salts are more inhibitory (Al Soudi et al. 2017). Current conditions at the near-surface of Mars may support deliquescence of certain perchlorate salts at discrete locations for brief intermittent periods (Nuding et al. 2014; Rummel et al. 2014; Fischer et al. 2016; Jänchen et al. 2016; Primm et al. 2017; Orosei et al. 2018; Rivera-Valentín et al. 2018, 2020; Nair and Unnikrishnan 2020; Pál and Kereszturi 2020). The salts examined here, except for perchlorates, form eutectic brines that require somewhat warmer conditions to remain liquid than observed currently at the surface of Mars. While perchlorate salts have the lowest eutectic temperatures among the Mars-relevant salts, these appear to be substantially more inhibitory to microbial growth and survival than chlorate, chloride, or sulfate salts (Al Soudi et al. 2017; Heinz et al. 2020).

Not all of the salts tested in the current study may be important determinants of habitability on Mars or the ocean worlds. The salts were chosen in part to create an iterative matrix of ions, enough for robust testing of models when coupled with the physical parameters of the salts and ions. Near the surface of Mars are the most important cations are Ca, Mg, K, Na and the most important anions are Cl, SO_4_ and (per)chlorates. Another important element on Mars is Fe, which for the bacteria tested in the current study, was toxic at mM concentrations, too low to appreciably affect *a*_*w*_. This may well have been due to the increasingly lower pH (to below pH 4) needed to solubilize the Fe minerals at higher concentrations. Nitrates and ammonium are sound sources of N for microbial growth, but may not form dense brines on Mars, although ammonia-water solutions might explain the liquid suggested under the south polar cap (Egea-González et al. 2025). Similarly, PO_4_ salts are likely not forming dense brines on Mars. Ocean worlds may have briny oceans rich in Mg and Na salts of Cl and SO_4_ (Fagents 2003; Ruiz et al. 2007; Brown and Hand 2013; Vance et al. 2014; Chivers et al. 2023). Ammonia may be important in the oceans of Ceres, Enceladus, Pluto, and dwarf planets of the Kuiper Belt, among others, since ammonia solutions have even lower eutectic points than saline brines (Brown and Hand 2013; Glein et al. 2015; Nimmo and Pappalardo 2016; De Sanctis et al. 2020).

The microbes present in spacecraft assembly facilities (SAFs) are the most likely to be transported to another world. Given that the clean rooms where spacecraft are built have low humidity, microbes tolerant to aridity and higher salinity from dried deposits of droplets may be expected, as certain studies have shown (Moissl et al. 2013; Venkateswaran et al. 2014; Smith et al. 2017). Our previous work showed that microbes tolerant to high MgSO_4_, (per)chlorates, and NaCl, represented 1 to 10% of the culturable heterotrophic microbial community in SAFs (Carte et al. 2020; Eberl et al. 2025). When microbial samples from an SAF were enriched at high salinities for 6 months, the resulting community contained members of all the guilds needed for functioning biogeochemical cycles (Carte et al. 2024). Individual polyextremophile microbes are unlikely to survive and proliferate for long periods of time, but tolerant microbial communities may persist, since key ecosystem functions could operate. Terrestrial field analogs and laboratory analogs are widely used to determine the limits of living systems and their functions, as in the current study (Hallsworth et al. 2021; Jung et al. 2022). None of the tolerances reported in the current study have set new records for the most important salts observed or expected on Mars or the ocean worlds. The lowest *a*_*w*_ studied here is still higher than the current records for growth in solutions with high solutes (Lockhead and Landerkin 1942; Ōnishi 1963; Pitt and Christian 1968; Tokouka and Ishiatani 1991; Stevenson et al. 2015, 2017). Accordingly, there is no impetus here to change the limits for the chemical conditions used to define the Special Regions where life is more plausible, previously established at an *a*_*w*_ of 0.5 (Rummel et al. 2014).

## Supplementary Information

Below is the link to the electronic supplementary material.

Supplementary material 1 (DOCX 97.3 kb)

## Data Availability

The datasets generated during the current study are available from the corresponding author on reasonable request.
