# Supplementary material for: The effects of brines relevant to Mars and the ocean worlds on bacterial growth reflect salt-specific responses across water activity
Source: Arch Microbiol. 2025 Sep 9;207(10):258. doi: 10.1007/s00203-025-04418-9 (PMC12420771; doi:10.1007/s00203-025-04418-9)
Supplement: Supplementary file 1 — Supplementary material 1 (DOCX 97.3 kb) [file 203_2025_4418_MOESM1_ESM.docx]

**Supplementary Data**

Table S1. Chemical parameters used for statistical models of Core Ions.

| Cation | Anion | Molarity | Molality | # ions | Osmotic Coefficient | Van 't Hoff Coefficient | Osmolarity | Osmotic Pressure | Water Activity | Ionic Strength | Percent Saturation |
| --- | --- | --- | --- | --- | --- | --- | --- | --- | --- | --- | --- |
| Magnesium | Chloride | 0.1 | 0.100 | 3 | 0.861 | 2.583 | 0.258 | 640.0 | 0.985 | 0.30 | 2 |
| Magnesium | Chloride | 1.0 | 0.977 | 3 | 1.101 | 3.303 | 3.303 | 8183.4 | 0.942 | 3.00 | 18 |
| Magnesium | Chloride | 2.0 | 1.900 | 3 | 1.482 | 4.446 | 8.892 | 22030.6 | 0.835 | 6.00 | 35 |
| Magnesium | Chloride | 3.0 | 2.793 | 3 | 1.921 | 5.763 | 17.289 | 42834.7 | 0.598 | 9.00 | 53 |
| Magnesium | Chloride | 4.0 | 3.615 | 3 | 2.345 | 7.035 | 28.140 | 69718.9 | 0.581 | 12.00 | 70 |
| Magnesium | Nitrate | 0.1 | 0.100 | 3 | 0.872 | 2.616 | 0.262 | 648.1 | 0.996 | 0.30 | 2 |
| Magnesium | Nitrate | 1.0 | 0.986 | 3 | 1.065 | 3.195 | 3.195 | 7915.8 | 0.971 | 3.00 | 20 |
| Magnesium | Nitrate | 1.5 | 1.432 | 3 | 1.183 | 3.549 | 5.324 | 13189.4 | 0.964 | 4.50 | 31 |
| Magnesium | Nitrate | 2.0 | 1.860 | 3 | 1.305 | 3.915 | 7.830 | 19399.4 | 0.907 | 6.00 | 41 |
| Magnesium | Nitrate | 3.0 | 2.742 | 3 | 1.620 | 4.860 | 14.580 | 36123.0 | 0.886 | 9.00 | 61 |
| Magnesium | Sulfate | 0.1 | 0.100 | 2 | 0.550 | 1.100 | 0.110 | 272.5 | 0.989 | 0.40 | 4 |
| Magnesium | Sulfate | 1.0 | 0.993 | 2 | 0.527 | 1.054 | 1.054 | 2611.4 | 0.978 | 4.00 | 36 |
| Magnesium | Sulfate | 2.0 | 1.952 | 2 | 0.670 | 1.340 | 2.680 | 6639.9 | 0.940 | 8.00 | 71 |
| Magnesium | Sulfate | 2.8 | 2.686 | 2 | 0.838 | 1.676 | 4.693 | 11626.7 | 0.978 | 11.20 | 100 |
| Potassium | Chloride | 0.1 | 0.100 | 2 | 0.927 | 1.853 | 0.185 | 459.1 | 0.995 | 0.10 | 2 |
| Potassium | Chloride | 1.0 | 0.970 | 2 | 0.897 | 1.794 | 1.794 | 4444.8 | 0.969 | 1.00 | 21 |
| Potassium | Chloride | 1.5 | 1.436 | 2 | 0.899 | 1.798 | 2.697 | 6682.0 | 0.954 | 1.50 | 32 |
| Potassium | Chloride | 3.0 | 2.752 | 2 | 0.930 | 1.860 | 5.580 | 13824.9 | 0.902 | 3.00 | 64 |
| Potassium | Chloride | 4.0 | 3.580 | 2 | 0.953 | 1.906 | 7.624 | 18889.0 | 0.890 | 4.00 | 85 |
| Potassium | Chloride | 4.7 | 4.152 | 2 | 0.969 | 1.938 | 9.109 | 22567.2 | 0.873 | 4.70 | 100 |
| Potassium | Nitrate | 0.5 | 0.489 | 2 | 0.820 | 1.640 | 0.820 | 2031.6 | 0.983 | 0.50 | 17 |
| Potassium | Nitrate | 1.0 | 0.956 | 2 | 0.762 | 1.524 | 1.524 | 3775.8 | 0.977 | 1.00 | 33 |
| Potassium | Nitrate | 1.5 | 1.406 | 2 | 0.720 | 1.440 | 2.160 | 5351.6 | 0.973 | 1.50 | 50 |
| Potassium | Nitrate | 2.0 | 1.834 | 2 | 0.685 | 1.370 | 2.740 | 6788.5 | 0.966 | 2.00 | 67 |
| Potassium | Nitrate | 3.0 | 2.741 | 2 | 0.619 | 1.238 | 3.714 | 9201.7 | 0.962 | 3.00 | 100 |
| Potassium | Sulfate | 0.1 | 0.100 | 3 | 0.779 | 2.337 | 0.234 | 579.0 | 0.997 | 0.30 | 13 |
| Potassium | Sulfate | 0.8 | 0.777 | 3 | 0.657 | 1.971 | 1.577 | 3906.6 | 0.981 | 2.40 | 100 |
| Sodium | Chloride | 0.1 | 0.100 | 2 | 0.924 | 1.848 | 0.185 | 457.9 | 0.989 | 0.10 | 2 |
| Sodium | Chloride | 1.0 | 0.979 | 2 | 0.939 | 1.878 | 1.878 | 4652.9 | 0.973 | 1.00 | 16 |
| Sodium | Chloride | 2.0 | 1.927 | 2 | 0.980 | 1.960 | 3.920 | 9712.1 | 0.901 | 2.00 | 32 |
| Sodium | Chloride | 3.0 | 2.813 | 2 | 1.028 | 2.056 | 6.168 | 15281.7 | 0.883 | 3.00 | 48 |
| Sodium | Chloride | 4.0 | 3.673 | 2 | 1.100 | 2.200 | 8.800 | 21802.6 | 0.852 | 4.00 | 65 |
| Sodium | Nitrate | 0.1 | 0.100 | 2 | 0.918 | 1.836 | 0.184 | 454.9 | 0.994 | 0.10 | 1 |
| Sodium | Nitrate | 0.5 | 0.493 | 2 | 0.870 | 1.740 | 0.870 | 2155.5 | 0.972 | 0.50 | 5 |
| Sodium | Nitrate | 1.0 | 0.969 | 2 | 0.848 | 1.696 | 1.696 | 4202.0 | 0.970 | 1.00 | 10 |
| Sodium | Nitrate | 1.5 | 1.427 | 2 | 0.836 | 1.672 | 2.508 | 6213.8 | 0.966 | 1.50 | 14 |
| Sodium | Nitrate | 2.0 | 1.887 | 2 | 0.826 | 1.652 | 3.304 | 8185.9 | 0.965 | 2.00 | 19 |
| Sodium | Nitrate | 3.0 | 2.743 | 2 | 0.814 | 1.628 | 4.884 | 12100.5 | 0.940 | 3.00 | 28 |
| Sodium | Nitrate | 4.0 | 3.545 | 2 | 0.805 | 1.610 | 6.440 | 15955.6 | 0.887 | 4.00 | 38 |
| Sodium | Sulfate | 0.1 | 0.100 | 3 | 0.797 | 2.391 | 0.239 | 592.4 | 0.990 | 0.30 | 7 |
| Sodium | Sulfate | 1.0 | 0.986 | 3 | 0.640 | 1.920 | 1.920 | 4756.9 | 0.971 | 3.00 | 71 |
| Sodium | Sulfate | 1.5 | 1.440 | 3 | 0.612 | 1.836 | 2.754 | 6823.2 | 0.975 | 4.50 | 100 |

Table S2. Chemical parameters for modeling of All Ions.

| Cation | Anion | Molarity | Water Activity |
| --- | --- | --- | --- |
| Ammonium | Chloride | 1.0 | 0.98 |
| Ammonium | Chloride | 2.0 | 0.94 |
| Ammonium | Sulfate | 1.0 | 0.97 |
| Ammonium | Sulfate | 2.0 | 0.95 |
| Cesium | Chloride | 0.1 | 0.99 |
| Cesium | Chloride | 0.5 | 0.98 |
| Cesium | Chloride | 1.0 | 0.97 |
| Iron | Chloride | 0.01 | 0.99 |
| Magnesium | Chlorate | 0.1 | 1.00 |
| Magnesium | Chlorate | 1.0 | 0.99 |
| Magnesium | Perchlorate | 0.1 | 0.99 |
| Magnesium | Perchlorate | 0.5 | 0.97 |
| Magnesium | Perchlorate | 1.0 | 0.96 |
| Potassium | Chlorate | 0.1 | 0.99 |
| Potassium | Chlorate | 0.5 | 0.98 |
| Potassium | Chlorate | 1.0 | 0.98 |
| Potassium | Perchlorate | 0.1 | 0.99 |
| Potassium | Perchlorate | 0.5 | 0.98 |
| Potassium | Perchlorate | 1.0 | 0.97 |
| Potassium | Phosphate | 0.1 | 1.00 |
| Potassium | Phosphate | 1.0 | 0.98 |
| Sodium | Borate | 0.1 | 0.99 |
| Sodium | Borate | 0.5 | 0.99 |
| Sodium | Chlorate | 0.1 | 0.99 |
| Sodium | Chlorate | 0.5 | 0.98 |
| Sodium | Chlorate | 1.0 | 0.96 |
| Sodium | Chlorate | 1.5 | 0.94 |
| Sodium | Chlorate | 2.0 | 0.91 |
| Sodium | Chlorate | 2.7 | 0.89 |
| Sodium | Perchlorate | 0.1 | 0.99 |
| Sodium | Perchlorate | 0.5 | 0.98 |
| Sodium | Perchlorate | 1.0 | 0.96 |
